# The Effect of 8-Methoxypsoralen on Pituitary-Gonad Axis and Ovarian Function in Mice

**Published:** 2013-08-24

**Authors:** Esmail Fattahi, Seyed Gholam Ali Jorsaraei, Mossa Gardaneh, Eisa Tahmasbpour Marzony

**Affiliations:** 1 Department of Biology, Islamic Azad University, Ayatollah Amoli Branch, Amol, Iran; 2 Department of Anatomy and Embryology, Fatemeh Zahra Fertility, Infertility and Health Reproductive Research Center, Babol University of Medical Sciences, Babol, Iran; 3 Department of Molecular Genetics, National Institute of Genetic Engineering and Biotechnology, Tehran, Iran; 4 Department of Biology, Faculty of Basic Sciences, Azad University of Sari, Sari, Iran

**Keywords:** 8-Methoxypsoralen (8-MOP), LH, FSH, Ovary

## Abstract

**Objective:**

8-Methoxypsoralen (8-MOP) is a photoactive compound widely used in the treatment of proliferate disorders. The present study investigates the effects of 8-MOP on ovary function and pituitary-gonad axis in mice.

**Materials and Methods:**

: In this experimental analytical study, 45 female Balb/C mice were divided into three groups (n=15), control, sham (olive oil injection) and experimental. The experimental group were received an intraperitoneal (i.p.) injection of the LD50 dose of 60 mg/kg 8-MOP. At 30 days after injection, the animals were sacrificed while in the proestrus stage and examined for morphological and histological changes their ovaries. Blood samples were collected and estrogen, luteinizing hormone (LH) and follicle stimulating hormone (FSH) levels were assessed by radioimmunoassay. Data were analyzed using one-way ANOVA and the t test.

**Results:**

The mean levels of estrogen and progesterone in the experimental group significantly decreased (p< 0.001). However, there was a significant increase in LH and FSH levels in this group compared to the control groups (p< 0.001). The mean number and diameter of the corpus luteum (CL) and the number of growing follicles in the experimental group significantly reduced compared to the control and sham groups (p< 0.001). The mean granulosa thickness in the experimental group also significantly decreased compared to the control and sham groups (p< 0.001).

**Conclusion:**

Our data indicated that 8-MOP can affect the levels of LH, FSH, estrogen and progesterone. Our findings further suggest that consecutive doses of 8-MOP may impair the female reproductive tract (or development).

## Introduction

thoxypsoralen (8-MOP) is a photoactive synthetic metabolite produced in a variety of crop plants, in particular Ammi majus. It belongs to a group of drug compounds known as psoralens (1, 2). Psoralens when combined with long wave ultraviolet (UVA) light are commonly used to treat a wide range of skin disorders such as psoriasis, vitiligo and eczema (3-7). Psoralens can produce free radical, singlet oxygen and reactive oxygen species (ROS) such as oxygen superoxide anion. ROS production in cells is associated with a combination of psoralens and proteins or nucleic acids which show the biological therapeutic effects of psoralens. Analogues of psoralens can arrest the cell cycle in the S-phase (8-10). Although the mechanism of 8-MOP is not well known, its interaction with DNA has been demonstrated. During photoactivation, methoxsalen may cross-link with single strand or double strand DNA leading to deficiencies in DNA replication and inactivated gene expression (11-13). Psoralens alone or in combination with UVA radiation are capable of exerting cytotoxic effects such as inducing apoptosis and inhibiting cell proliferation (14). Parivar et al. (15) have shown significant anomalies including a reduced number of hepatocytes, glomerulus and renal tubules and increased number of megacaryocytes and nucleated erythrocytes in experimental animal embryos. Psoralens may cause skin and lung cancers, sister chromatic exchange, and chromosomal changes in humans (16-18). Some studies have shown that 8-MOP may cause reductions in fertilization, the number of corpus lutea (CL), uterine weight and implantation sites, and induce the expression of some liver enzymes at the mRNA level (19). According to a number of researches, some methoxsalen derivatives such as 5-methoxypsoralens reduce the fertilization rate. In addition, injection of this substance at a dose of 75 to 150 mg/kg causes atrophy in the pituitary gland and reduced sperm counts (20). Studies by Diawara et al. have demonstrated that 8-MOP can cause reductions in the pituitary gland, vesicle seminal, prostate, testis and epididymis weights and increase liver weight (20).

Based on available reports on the adverse effects of 8-MOP, this investigation aimed to study the effects of 8-MOP on the structure of ovarian tissue and changes in sex hormone levels in mice.

## Materials and Methods

### Preparation of laboratory animals

In this experimental-analytical study, adult female Balb/C mice, with a weight range of 30- 35 g and 70-80 days of age were obtained from Pasteur Institute (Experimental Animal Keeping, Center, Tehran, Iran). They were randomly divided into three groups: experimental (n=15), control (n=15) and sham (n=15). The animals were maintained in standard cages at 25 ± 2˚C under 12 hour light: 12 hour dark conditions, with access to powdered diet and deionized water. Before starting the experiment, all mice were sexually co-cycled in proestrus. Vaginal smears were used to determine the appropriate cycle. In order to prepare the vaginal smear, the vaginas were washed by saline and then spread on a slide. Smears were stained with giemsa and evaluated under a light microscope for cycle identification (21).

### Preparation of 8-methoxypsoralen

We dissolved 0.04 g of 8-MOP (Sigma Aldrich, USA) in 1.5 ml olive oil to make a stock solution of 0.026 g/ml. Commercial 8-MOP was injected intraperitoneally in a single dose (60 mg/kg) based on its LD50 .

### Treatment with 8-methoxypsoralen 

On the evening of day 1, each animal in the experimental group received an intraperitoneal (i.p.) injection of a single dose of 8-MOP (60 mg/kg). Injections were administered for five consecutive days each week for a period of one month. The sham group received the same volume (60 mg/kg) of Olive oil and the control group received no injections. All animal-related protocols were approved by the Ethical Committee at Babol University of Medical Sciences, Babol, Iran.

### Tissue processing and morphological observation

At 24 hours following the final injection, animals were sacrificed by ether and their tissues prepared for morphological and histological examinations. The animals’ ovaries were removed and fixed in 10% formalin for at least 48 hours. Serial tissue sections that were 5 µm in diameter were prepared by using a microtome (Easy cut, Diapath, Italy). For histological processing, the sections were examined under a light microscope for morphological and histological parameters that included the numbers and diameters of CL and granulosa thicknesses by calibrated graticule.

### Hormonal assays

Blood samples were collected from each animal’s underarm area, then centrifuged at 3000 rpm for 15 minutes until the serum was separated. Serum concentrations of luteinizing hormone (LH) and follicle stimulating hormone (FSH), estrogen and progesterone were measured by a radioimmunoassay method with special animal kits (USCN, China).

### Statistical analysis 

Data are reported as means ± SD. We used the independent t test and one-way ANOVA to compare means between the groups. Probability p value less than 0.05 were considered statistically significant.

## Results

### Hormone concentrations 

Table 1 shows the mean values of the measured hormones for all three groups. The mean ± SD of LH in the 8-MOP group was 4.5 ± 0.49 IU/L, for the control group it was 3.3 ± 0.53 IU/L and the sham group had a mean LH value of 3.4 ± 0.51 IU/L. There was no significant difference observed in the mean LH values between the control and sham groups, whereas this difference was significant when compared to the experimental group (p< 0.05).

The mean ± SD levels for FSH were as follows for the experimental (4.7 ± 0.89 IU/L), control (3.5 ± 0.5 IU/L) and sham (3.58 ± 0.47 IU/L) groups. There was a non-significant difference noted in the mean FSH values between the control and sham groups, however the difference was significant when compared to the experimental group (p<0.05).

Both the estrogen and progesterone mean levels for the experimental group showed a significant decrease compared to the control and sham groups (p<0.001). There was no significant difference observed between in these levels between the control and sham groups (Table 1).

### The number and diameter of corpora lutea (CL) 

Table 2 shows the mean value for the number and diameter of CL in all three groups. The mean number and diameter of CL in the experimental group were significantly decreased compared to the control and sham groups (p<0.001). However this difference was not significant between the control and sham groups (Table 2).

### Number of growing follicles 

In this study, i.p. injections of 8-MOP significantly reduced the number of growing follicles in the experimental group compared to the control and sham groups (p<0.001; Table 2).

**Table 1:** The effects of 8-methoxypsoralen (8-MOP) on hormonal concentrations in the groups


Parameters	Control	Sham	Experimental	P value

**LH (IU/L)**	3.3 ± 0.534	3.4 ± 0.51	4.5 ± 0.49	<0.05
**FSH (IU/L)**	3.5 ± 0.5	3.58 ± 0.47	4.7 ± 0.89	<0.05
**Progesterone (pg/ml)**	156 ± 4.32	154 ± 4.23	113.2 ± 2.4	<0.001
**Estrogen (pg/ml)**	255 ± 6.24	253 ± 5.09	145 ± 6.78	<0.001


**Table 2:** Mean number and diameter of corpora lutea (CL), granulosa thickness and number of growing follicles


Parameters	Control	Sham	Experimental	P value

** of CL (µm)**	643.97 ± 5.95	640.86 ± 5.2	456.86 ± 6.2	<0.001
**Number of CL**	5.32 ± 0.261	5.1 ± 0.252	3.86 ± 0.21	<0.001
**Granulosa thickness (µm)**	50.46 ± 0.1	48.36 ± 0.13	31.76 ± 0.131	<0.001
**Number of growing follicles**	10.33 ± 0.23	10.27 ± 0.25	7.19 ± 0.28	<0.001


### Granulosa thickness

**Fig 1:**
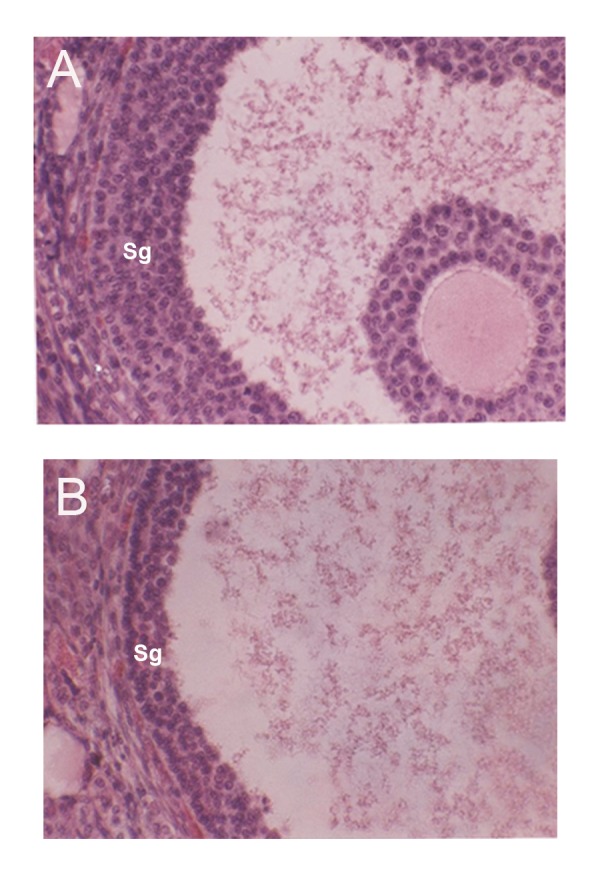
A comparison of the stratum granulosa (sg) from ovarian tissue in the control (A) and experimental (B) groups. Granulosa thickness in the experimental group was significantly lower than the control group.

The mean granulosa thicknesses in all three groups are shown in table 2. There was a significant decrease in the mean granulosa thickness in the experimental group compared to the control and sham groups (p<0.001; Fig 1). This difference was not significant between the control and sham groups.

## Discussion

In this study i.p. injections of 8-MOP into a mice model has resulted in reductions in the number and diameter of CL, decreased granulosa thickness and decreased numbers of growing follicles. Several studies have shown a negative role for 8-MOP in crosslinking with DNA strands-a reaction that promotes inhibition of DNA replication. Therefore, mitotic division may be inhibited upon 8-MOP administration and the normal replacement of progenitor cells can be halted or delayed (10- 12). 

Psoralens are usually produced during normal metabolism in plants, particularly in many fruits and vegetables. Some studies have reported these elements have the capability to reduce birth rate and block embryo implantation. Psoralens can also inhibit CL development in the ovaries, reduce uterine weight and disrupt estrogen cycling (19, 22).

8-MOP can produce ROS and free radical during the photoactivation process, therefore accelerating oxidation of DNA, lipids and proteins (23, 24). These events lead to reductions in the number and diameter of CL (25). Several studies have reported that 8-MOP dose-dependently decreases CL number, implantation rate, percentage of birth rate and testosterone levels (19, 20).

In the current study, there was a significant decrease in the mean CL diameter in the experimental group, which has indicated that CL underwent degeneration as a result of the destructive function of 8-MOP. Since the number of CL is dependent on the number of oocytes, a reduction in number of growing oocytes can lead to a reduced number of CL. In our study, we have shown that the mean estrogen levels in the experimental group significantly decreased. Estrogen is secreted from the granulosa cells. Reduction in the number of CL is directly dependent on the released oocytes (19). Therefore, it can be inferred from this relationship that follicle growth and subsequent release of oocytes diminish due to the use of methoxsalen. Additionally, after ovulation the remaining follicle cells convert to CL that begin to secrete progesterone. In the current study, the reduced number of follicles detected have led to reduced granulosa cells and consequently reduced progesterone secretion.

Due to the reduction in the number and diameter of CL, presumably the secretion of progesterone has been disturbed. Studies have shown that methoxsalen induces the generation of ROS that may disturb mitochondrial function and induce apoptosis by activating caspases 3, 8 and 9 (26). These findings support the hypothesis that either parallel or subsequent to a reduction in the number of CL, a reduction in progesterone secretion will also occur. Our data have shown a reduction in the diameter of the CL, a change that can occur due to atrophication of CL cells and their inability to secrete hormones. Therefore, the reduced levels of progesterone observed in the current study favor this hypothesis.

Once affected by compounds such as methoxsalen, CL cells lose their normal function. Changes such as reduced cell diameter, atrophy or death of CL cells eventually lead to reduced levels of estrogen and progesterone. Diawara and colleagues have shown that 8-MOP and 5-MOP decrease estrogen levels. Estrogen is synthesized by aromatase, a CYP450 enzyme, within the granulosa. Data from several studies have indicated that methoxsalen significantly reduces the level of aromatases and estradiol (19, 22). Psoralens can induce microsomal cytochrome oxidase CYP1A1 *in vitro*. Some studies indicate that psoralens reduce estrogen levels by inducing enzymes such as CYP1A1 and CYP1A2 (27). These enzymes play an important role in hydroxylation of estrogen. Gwang et al. have reported the 8-MOP can induce hepatic CYP1A1 and i.p. injection of 8-MOP induces expression of CYP2B and CYP1A mRNA in addition to elevating their catalytic activities (28). 8-MOP can induce the Ah receptor that is associated with CYP1A2 enzyme induction. CYP1A2 decreases blood estradiol levels by catalyzing estradiol to 2-hydroxy estradiol which eventually causes reductions in ovulation and fertilization rates (29).

The reduction in granulosa cell numbers observed in 8-MOP-injected animals suggested that reduction of estradiol might be another result of 8-MOP function. Some studies have shown that methoxsalen results in decreased levels of steroid hormones by inducing their catabolism (19, 22). Considering the role of estrogen in follicular development (30), the decreased levels of this hormone observed in the experimental group might reduce follicular growth and development, and exert a negative effect on ovulation.

It is clear from our results that i.p. injection of methoxsalen has resulted in elevated LH and FSH hormones. These two hormones are regulated by the hypothalamus that produces small peptide hormones called releasing factors. When the estrogen level is low, gonadotropin-releasing hormone (GnRH) secretion from the hypothalamus is induced via a feedback mechanism that ultimately induces LH and FSH production and release. LH and FSH impact the ovaries to produce estradiol and progesterone. Since estradiol has a negative feedback effect on the hypothalamus-pituitary axis, its secretion results in inhibition of GnRH release and LH/ FSH production (31, 32). In this study estradiol and progesterone levels have been reduced, thus we conclude that estrogens may not have a negative feedback effect on the hypothalamus-pituitary axis and may not inhibit production of these hormones. LH receptors in growing follicles reside in the theca cells, whereas FSH receptors are located in granulosa cells. One result of the production of free radicals and ROS by methoxsalen (33, 34) is peroxidation of the membrane lipid in luteal cells which may cause loss of receptors for the gonadotropins, and ultimately reduce the steroid of the CL due to the atrophication process (35, 36). LH and FSH cause increased numbers and growth of follicles (37). However in the current study gonadotropin increased but the number of growing follicles reduced. Methoxsalen might act directly on ovarian tissue and cause a decreased number of follicles in the ovaries. 

## Conclusion

In the present study, we have shown that 8-MOP decreases progesterone secretion by reducing the number and diameter of CL, thus the uterus would not be ready for implantation. The decreased number and diameter of CL are associated with reductions in the rate of follicle growth and ovulation. Our results suggest that 8-MOP can alter hormone production by exerting a negative effect on ovarian tissue or the central nervous system. Results of the present study have shown that 8-MOP interferes with the production of reproductive hormones in female mice and, therefore, adversely affect fertilization. 
